# Reproductive health service utilization and associated factors: the case of north Shewa zone youth, Amhara region, Ethiopia

**DOI:** 10.11604/pamj.supp.2016.25.2.9712

**Published:** 2016-11-26

**Authors:** Wassie Negash, Muluken Dessalegn, Berhanu Yitayew, Mohammed Demsie, Maereg Wagnew, Josephat Nyagero

**Affiliations:** 1 Institute of Medicine and Health Science, College of Health Science/Medicine, Debre Berhan University, Debre Berhan, Ethiopia; 2 Amref Health Africa in Ethiopia, Monitoring Evaluation and Research Department, Addis Ababa, Ethiopia; 3 College of Social Science, Department of Psychology Debre Berhan University, Debre Berhan, Ethiopia; 4 Maternal and Child Health Directorate, FMOH, Ethiopia; 5 Amref Health Africa, HQ, Kenya

**Keywords:** Youth, reproductive health service, utilization cross sectional, regression

## Abstract

**Introduction:**

Many youth are less informed, less experienced and less comfortable in utilizing reproductive health services. In the Sub-Saharan region the adolescents account for a higher proportion of new HIV infections and unmet need for reproductive health (RH) services. This study assessed reproductive health service utilization and associated factors among the youth in Amhara Region, Ethiopia.

**Methods:**

A community based cross-sectional study was conducted from June 15-July 30, 2014. Three hundred ninety one youth were selected by systematic random sampling technique and interviewed using structured questionnaire. Data were anlyzed using SPSS windows version 20. Multiple logistic regression was done to control potential confounding variables. P-values <0.05 were considered statistically significant.

**Results:**

Three hundred and nighty one in-school and out-of-school youth were interviewed; 256 (65.5%) participants were in school and 209 (53.5%) were males. Almost all respondents (93.9%) had heard about reproductive health services and a third 129 (33%) had ever practiced sexual intercourse and 54.7% of them had utilized at least one reproductive health services. Never had sexual intercourse (AOR=3.693, 95%CI: 1.266, 10.775), families that asked their children about friends (parental monitoring) (AOR=1.892, 95%CI: 1.026, 3.491), know where service provided (AOR=3.273, 95%CI: 1.158, 9.247), youths who reads newspaper readers (AOR=3.787, 95%CI: 1.849were independent predictors of youth reproductive service utilization at 95 % CI and p-value <0.05%.

**Conclusion:**

Even though the youth have information about reproductive health services, youth reproductive health services utilization is very low. Therefore, building life skill, facilitating parent to child communication, establishing and strengthening of youth centres and increasing awareness for youth about those services are important steps to improve adolescents' reproductive health (RH) service utilization.

## Introduction

World Health Organization defines the youth as persons within the age group of 15-24 years [1]. It is the period of adolescence or adulthood and characterized by physical, mental, emotional and social development [2]. Adolescence is the period of experimentation and engagement of a wide range of behaviours that place their life at high risk [3]. And they are more than 20% of the world’s population, of whom 85% live in developing countries [4–6]. Many reports show that sexual activity, early pregnancies and sexually transmitted infections (STIs) including HIV infection rates are increasing among youths mainly in developing countries [7]. Up to 100 million youths become infected with a curable sexually transmitted disease and about one out of 20 youths in the world contract an STI each year [5, 6, 8].

The utilization of reproductive health services is an important component in preventing adolescents from different sexual and reproductive health problems. According to the 2011 Ethiopian Demographic and Health Survey, contraceptive use among currently married women of 15–19 years of age was only 23%, with 0% utilization of permanent methods, 1.6% and 2.5% utilization of implants and IUD, respectively. Contraceptive use was lower when compared with other age groups. Among sexually active youth aged 15–19 years, women and men who were tested for HIV test were only 24% and 27% respectively [7]. Therefore, assessing factors affecting reproductive health service utilization within this age category, especially FP and VCT services, are very important to improve adolescent reproductive health service utilization and thereby reduce the burden of adolescent disease and disabilities associated with RH.

The Ethiopian government developed national adolescent and youth reproductive health strategy. Besides in the Health Extension Program (HEP) (2004) adolescence reproductive health were included to promote adolescent reproductive health services in urban and ruler area. Despite these efforts, there is little information about RH utilization and factors associated with RH utilization in the rural area. Therefore, this study assessed youth’s reproductive health utilization in rural setting and factors that affect its utilization. In addition it put baseline for emphasis on social factors that affect or hinder utilization of services and plan on those factors to bring behavioural change as well as reaching rural community with full range of reproductive health services.

## Methods

### Study design, area and population

A community-based cross-sectional study using both qualitative and quantitative data collection method was conducted in North Shewa, Amhara region from June15 to July 30, 2014. Males and females, 14 to 24 years of age, who were residing in one of the randomly selected five districts of North Shewa zone, were eligible to participate in this study. North Shewa has 24 districts of which 3 urban and 21 rural districts. The major ethnic groups of the zone are Amhara. The population is predominantly Orthodox-Christian by religion followed by Muslim. Agriculture is the main livelihood of the population.

### Sample size and sampling procedure

EPI INFO window version 3.5.1 was used to calculate the sample size using single population proportion formula based on an assumption of proportion of reproductive health service utilization by taking 50% proportion; with marginal error of 5%, a standard score corresponding to 95% certainty, and nonresponse rate 2% since people are cooperative we took less nonresponse rate. The calculated total sample size comprised a total of 391 youths aged 14-24 years. Five districts were randomly selected from 24 districts in the Zone. For five districts, the list of kebele (lowest administrative structure in Ethiopia) with youth list was obtained with the support of the health extension worker in the community for sampling of the youths. Then, all youth aged between 14-24 years listed both from in and out of school to get the sampling frame. Then study subject (youth) were selected by systematic sampling technique at each five interval. For the proper representations of the sample in urban –rural the sampling frame were stratified in to two at beginning

### Data collection and analysis methods

The selected 391 youth were then interviewed using structured pre-tested questionnaire. Besides, six key informants were interviewed and four FGD were done. Data were entered with EPI-data version 3.1 and cleaned and analyzed using SPSS version 20. Frequencies and cross tabulations were used to summarize descriptive statistics of the data and tables and graphs were used for data presentation. Bivariate analysis was done primarily to check which variables have association with the dependent variable. Then those variables have p-value ?0.1 in the bivariate analysis were entered in to Multiple Logistic regression. Finally the variables which had significant association at 95% CI and p-value?0.05 were identified as independent predictors of youth service utilization. Qualitative data was transcribed, edited, and thematically analysed. Then results were triangulated with quantitative results.

### Ethical considerations

The study was approved by the Institutional Ethical Review Board (IRB) of the Faculty of Medicine, Debre Berhan University and Amref Health Africa approved the proposal. Informed verbal consent was obtained from the youths and for their family when necessary for approval. Confidentiality and privacy were maintained during data collection, analysis and reporting.

## Results

### Socio-demographic characteristics

A total of 391 youth in and out of school participated in the study with a 92.7% response rate, of which 53.5 were male and 46.5% were female. The mean age was 19 years. Most respondents (76.7 %) were not married (Table 1).

**Table 1:** Socio-demographic characteristics of respondents in selected districts of North Shewa zone, 2014

Variable		Frequency (N=391)	Percent (%)
Sex	Male	209	53.5
	Female	182	46.5
Age *(Mean 19 years old)*	≤19 years	245	62.7
	>19 years	146	37.3
Marital status	Married	91	23.3
	Not- married	300	76.7
Religion	Orthodox	369	94.4
	Muslim	13	3.3
	Others (Protestant and catholic)	9	2.3
Schooling status	In School	256	65.5
	Out of School	135	34.5
Educational status	Cannot read and write	23	5.9
	Read and write	89	22.8
	Grade 1-8	43	11
	Grade ≥9	236	60.4
Mothers educational status	Cannot read and write	136	34.8
	Read and write	162	41.4
	Grade 1-8	39	9.9
	Grade ≥9	54	13.8
Fathers educational status	Cannot read and write	88	22.5
	Read and write	185	47.3
	Grade 1-8	39	10
	Grade ≥9	79	20.2

### Youth sexual intercourse experience or sexual history

Concerning sexual history, 150(38.4%) respondents had ever engaged in sexual intercourse and 163(38.6%) had sexual partners - of which nine in every ten, 151(92.6%) had one sexual partner and 12(7.4%) had multiple sexual partners. According the FGD discussions, one of the participants said, having multiple reasons was mentioned. Based on the discussant the purpose was to satisfy immediate sexual needs and for money. One of the saying among youths raised during the discussion was; when female youth asked by male for sexual intercourse and replied by another question as ‘do you have four legs or two legs?. This is referring to the wealth of males and to mean ‘Four legs (car) for very good wealth where as two legs for two feet don’t have money.

### Parental control

The relationship among the youth and their families is also important for RHS use. Even though it is not continuous, most parents ask their children about how (74.4%), where (78%), and with whom they spent their time (61%). More than half (55%) of youth reported that their families ask about where they spent their time. Among the study participants 65% of youths ask permission from their families to go out in the nighttime. Interviews and focus group discussions revealed that there was less parental follow up for their children. Most of the youth, especially students were living in rented houses. Due to this reason, there is not much parental control. So youth went to other places instead of schools. Youths spend their free time in different places, some of which were risky environments for example night clubs, chewing chat

### Risk perception for RH problems

Almost a half of the adolescents (49.1%) believed that they were at risk of RH problems. Most feared HIV (79.2%), unwanted pregnancy (32.3%) and/or STI (28.1%). Results from the FGDs and key informants revealed that the youth were chewing ‘chat’, smoking cigarettes, smoking hashish, drinking alcohol, and practicing unsafe sex. As a result, the youth are at risk of HIV/STI infection and unwanted pregnancy.

### Reproductive health service utilization

Almost all respondents (93.9%) had heard about reproductive health services (RHS). The most common sources of information were health workers and radio (Table 2). Slightly more than half of the respondents had utilized RHS of which 79.9 % were used within the past 12 months. Health centres and health posts were the most locations for youth reproductive health services. Still there are considerable numbers of youth who are not using RHS due to many different reasons (feel afraid, lack of preferred services, fear of side effects, inconvenient service hour, lack of knowledge about RHS, lack of privacy, religious opposition, judgemental attitude of health workers, opposition from sexual partner and cost of services were the major reasons in descending order). In the FGD, there was consensus that almost all youth had enough information about reproductive health. Some of the services are guidance and counselling services, peer education, HIV/STI screening, and FP services. But youths are not considering the potential consequences of the activities they are engaging in and they have low service-seeking behaviour. The youth prefer not to use long-acting FP methods for various reasons. Besides, youth who had previous risky behaviour were often not willing to be tested for HIV because they did not want to know their status during a fear of test positive (Table 2).

**Table 2:** Sexual reproductive health service information and service utilization of youths in North Shewa Zone, 2014

Variables		RHS (N=391) n (%)	FP (N=391) n (%)	STIs (N=391) n (%)	VCT (N=391) n (%)
Have you ever heard about …?	Yes	367 (93.9)	374 (95.7)	360 (92.1)	366 (93.8)
	No	24 (6.1)	17 (4.3)	31 (7.9)	25 (6.2)
What were your sources of information?^1^	Radio	179 (45.8)	NA	248 (63.7)	247 (63.3)
	TV	153(39.1)	223 (57.2)	203(52.1)	210(53.8)
	Family/relatives	90(23)	105(26.9)	112(28.7)	99(25.4)
	Newspaper/magazine	74(18.9)	99(25.4)	107(27.4)	103(26.4)
	Friends/peer group	120(30.7)	126(32.2)	139(35.6)	141(36.2)
	Teachers	112(28.6)	139(35.6)	163(41.8)	155(39.7)
	Pamphlet/ posters	87(22.3)	95(24.4)	120(30.8)	121(30.9)
	Health workers	206(52.7)	208(53.3)	263(66.9)	247(63.2)
	Others^2^	22(5.6)	20(5.3)	12(3.1)	15(3.8)
Have you ever discussed with others?	Yes	(NA)	293 (75.1)	311 (79.5)	296 (75.9)
Who did you discuss this with?^1^	Peer group	NA	126 (32.2)	175 (44.9)	167 (42.8)
	Parents	NA	118 (30.2)	138(35.4)	128(32.8
	Teacher(s)	NA	64(16.4)	143(36.7)	119(30.5)
	Sexual partner	NA	82(21)	134(34.8)	117(30)
	Others^2^	NA	39(10)	34(8.7)	36(9.2)
Have you ever utilized the services available?	Yes	214 (54.7)	156 (40)	79 (20.3)	281 (72.1)
	No	177 (44.8)	235 (60)	3 (17.2)	109 (27.9)
From where did you get the services	Health center	183(85.5)	75(19.2)	64(16.5)	NA
	Health post	152(71)	72(18)	NA	NA
	Private clinic	91(42.5)	36(9.7)	25(6.4)	NA
	Other^3^	16(7)	2	31(8)	NA

1 Participants could select more than one response

2 Health workers, training sessions, self-reading

3 Hospital, pharmacy, traditional healer

### Family planning utilization

Of the 391 participants, 374 (95.7) had heard about family planning (FP). Nine in every ten (90.8%) knew where FP services were available. About 75% of study participants had had discussions about FP, usually with their peers or parents. Only 109 (27.9%) and 113(29%) study participants used FP methods during their first and last sexual encounter, respectively. FP had been used by 40% of participants.

### Sexually transmitted infection service utilization

Majority of study participants (92.1%) had heard about STIs; the main sources of information were health workers and radio. Of those who knew where to go for STI diagnosis and treatment, health centers were found to be the primary location. About 311 (79.7%) of study participants had ever discussed about STIs. Majority (35.4%) of the study participants had discussed with their peers about STIs and taught in school by their teachers. Parents, sexual partners were also some who discussed about STI (Table 2).

The prevalence of STI symptoms in this study was also assessed: burning sensation during urination (20%), itching (8.2%), bad smell discharge (7.2%), genital ulcer (4.9%), and swelling (4.1%) of respondents were a reported symptoms from the participants. Of those participants who experienced these symptoms less than half (46%) of them ever sought diagnosis and treatment. Their reasons for not seeking STI diagnosis and treatment were feeling afraid, cost of services, inconvenient service hour from top to least.

### VCT service utilization

This study also assessed the voluntary counselling and testing (VCT) utilization among the youth. The majority (93.8%) had heard about VCT and the main sources of information were radio (63.3%) and health workers (63.2%). A high proportion (75.9%) had discussed VCT, most frequently with their peer group. A notable proportion (44.4%) considered themselves at risk for HIV and a high proportion (72.1%) had received VCT services (Table 2).

### Factors associated with youth reproductive health service utilization

In the multivariate logistic regression analysis, father’s educational status was significantly and independently associated with youth RHS utilization (Table 3). Participants with fathers who could read and write were more likely to use RHS than those whose fathers could not read and write (AOR=2.104, 95%CI: 1.070, 4.13). Sex of the respondent, marital status, schooling status and mother’s educational status were not independently associated with service utilization, although they were significantly associated with service utilization in the bivariate analysis. Utilization of RHS associated with use of newspapers as readers (AOR=3.787, 95%CI: 1.849, 7.75), families that asked their children about friends (parental control) (AOR=1.892, 95%CI: 1.026, 3.491), never had intercourse (AOR=3.693, 95%CI: 1.266, 10.775), knowledge of where RHS were provided (AOR=3.273, 95%CI:1.158,9.247), thought that they had no risk of RH problem (AOR=1.911, 95%CI: 1.130,3.233) (table3).

**Table 3:** Bivariate and multivariate analysis of factors associated with youth reproductive health utilization in North Shewa zone 2014

Variables	Utilization status		Crude OR (95% CI)	Adjusted OR(95% CI)
	Yes	No		
Sex of the respondent				
Male	81	128	1.00	1.00
Female	62	119	1.92 (1.412 2.609)	1.4(.825, 2.32)
Marital status of the respondent				
Married	43	48 198	1.00	1.00
Not married	100		1.774(1.1012.857)	1.15(.604, 2.19)
Schooling status				
Currently student	71	184	2.962(1.917 4.576)	0.58(.31, 1.06)
Currently not student	72	63	1.00	1.00
Mother’s educational Status				
Cannot read and write	50	85	1.00	1.00
Can read and write	67	95	834(.52, 1.33)	0.692 (.378, 1.27)
1-8	16	22	81(.389, 1.68)	0.624(.246, 1.578)
Grade 9& above	10	43	2.6(1.17, 5.47)	1.1(.364, 3.077)
Father’s educational Status				
Cannot read and write	45	43	1.00	1.00
Can read and write	63	122	2.3(1.209, 3.4)	2.104 (1.070, 4.139)^+^
1-8	17	21	1.29(.602, 2.8)	1.254(.466, 3.373)
Grade 9 & above	18	61	3.6(1.82, 6.942)	2.329(.822, 6.596)
Use Newspaper				
Yes	12	66	3.981(2.068, 7.662)	3.787(1.849, 7.755)
No	131	181	1.00	1. 00
How often do your families ask?				
Always	32	105	1.00	1.00
Sometimes	84	95	0.345(.211, .564)	0.513(.284, .929)^+^
Family ask your friends				
Yes	73	164	1.895(1.244, 2.886)	1.892(1.026, 3.491)^+^
No	70	83	1.00	1.00
Seek approval of your parents for night out				
Yes	82	173	1.763 (1.147, 2.709)	1.751(0.926, 3.3)
No	61	73	1.00	1.00
Ever had sexual partner				
Yes	79	84	1.00	1.00
No	63	163	2.395(1.571, 3.652)	2.010(0.966, 4.182)
Ever had sexual intercourse				
Yes	71	58	1.00	1.00
No	72	189	3.213 (2.069, 4.992)	3.693 (1.266, 10.775)^+^
Had sexual intercourse in last 12 months				
Yes	56	53	1.00	1.00
No	53	114	2.273(1.382, 3.737)	2.19(0.103, 0.984)^+^
Know where RHS are provided				
Yes	129	235	2.13 (0.96, 4.73)	3.273 (1.158, 9.247)^+^
No	14	12	1.00	1.00
Think that you have risk of RH problem				
Yes	86	108	1.00	1.00
No	57	139	1.94 (1.28, 2.95)	1.911(1.130, 3.233)^+^

+ Significantly associated at p<0.05

### Factors associated with family planning service utilization

As shown in Table 4, Bivariate analysis showed age of the respondent, ever had sexual partner, schooling status, Educational status, age of mother, age of father, father’s educational status, source of information, use TV, parental monitoring (family ask their friends), and marital status of the respondent were significantly associated with FP utilization. Whereas multivariate logistic regression analysis shows religion of the respondent, mother’s educational status, source of information use phone, those ever had sexual intercourse, or ever discussed about FP service are independent predictors of FP service utilization. Mothers who attended their education from grade one to eight (AOR=0.224, 95%CI: 0.068, 0.739) were less likely to utilize FP than those whose mothers could not read and write. Similarly those who are in school currently (AOR=.385, 95%CI: 1.032, 26.7) were less likely use FP than those out of school. Those who use phone as source of information use FP service 2.625 (AOR=2.625, 95%CI: 1.373, 5.016) times more likely than those not use phone. Those ever had no sexual intercourse use FP service 16.320(AOR=16.32, 95%CI: 6.27, 42.48) times more likely than those ever had. Those ever not discussed about FP service 5.074(AOR=5.074, 95%CI: 2.070, 12.440) times more likely use FP services than their counter parts (table 4).

**Table 4:** Bivariate and multivariate analysis of factors associated with FP utilization in North Shewa zone Ethiopia 20014

Variables	Utilization status		Crude OR (95% CI)	Adjusted OR(95% CI)
	Yes	No		
**age of the respondent**				
≤19	79	168	1.7(1.13,2.63)	**4.750(.953, 23.676)**
>19	64	79	1.00	1.00
**Marital status of the respondent**				
Married	61	30	1.00	1.00
Not married	94	206	4.46(2.70,7.35)	0.852(.376, 1.930)
**Religion of the respondent**				
Orthodox	194	220	1.00	1.00
Others	6	16	1.81(.69,4.72)	**5.251(1.032, 26.728)^+^**
**Schooling status**				
Currently student	65	191	5.88(3.73, 9.27).	**0**.**385(1.032, 26.7)**
Currently not student	90	45	1.00	1.00
**Educational status of the respondent**				
Cannot read and writ	14	9	1.00	1.00
Can read and write	39	50	1.99(0.78,5.09)	1.4 (0.35,5.84)
1-8	29	14	0.75(0.26,2.15)	0.411(0.08,2.15)
Grade 9 & above	73	163	3.47(1.44,8.389)	0.700(0.17,2.93)
**Mother’s educational status**				
Cannot read and write	57	59	1.00	1.00
Can read and write	67	95	1.02(0.64,1.62)	0.837(.398, 1.759)
1-8	21	17	0.58(0.28,1.21)	**0.224(.068, .739)^+^**
Grade 9& above	10	43	3.103(1.44,6.69)	0.623(.168, 2.315)
**Source of information Use TV**				
Yes	37	51	2.07(1.34,3.20)	1.458(0.693, 3.068)
No	85	100	1.00	1.00
**Use phone**				
Yes	15	24	2.13(1.41,3.22).000	**2.625(1.373, 5.016)^+^**
No	18	61	1.00	1.00
**Ever discussed about FP service**				
Yes	139	155	1.00	1,00
No	16	81	4.5(2.53,8.13)	**5.074(2.070, 12.440)**
**Ever had sexual intercourse**				
Yes	108	21	1.00	1.00
No	47	215	23.5(13.4,41.35).000	**16.320(6.27, 42.48)**

+ Significantly associated at p<0.05 %

### Factors associated with VCT service utilization

The result of bivariate analysis shows sex, age, marital status, schooling status of the respondent, mother’s educational status, age of father, father’s educational status, ever had sexual partner, ever discussed about VCT service, think of risk of HIV/AIDS, know where VCT is provided, ever had sexual intercourse significantly associated. While in multivariate logistic regression analysis shows age of the respondent is significantly associated with VCT service utilization in which adolescents age less than or equal to 19 years 2.25(AOR=2.25, 95%CI: 1.26, 4.02) times more likely use VCT services than those age20-24 years. Similarly participants whose mothers’ educational status Grade 9 & above were 4.9 (AOR=4.936, 95% CI: 1.6, 15.2) times more likely use VCT services than whose mothers cannot read and write. Meanwhile youths that never ever discussed about VCT 7.2 (AOR= 7.2, 95% CI: 3.693, 14.174) times more likely to use VCT service than ever discussed.

### Factors associated with STI service utilization

As presented in Table 5, in multiple logistic regression analysis those who ever had not sexual partner 3.8(AOR=3.8,95%CI:1.16, 12.4) times more likely use STI diagnosis and treatment services once they had sign symptoms of STI than those ever had sexual partner. Similarly, those ever discussed about STI with their teachers 0.25(AOR=.25,95%CI:.08, .75) times less likely use STI diagnosis and treatment services once they had sign symptoms of STI than their counter parts.

**Table 5:** Bivariate and multivariate analysis of factors associated with FP utilization in North Shewa zone Ethiopia 20014

Variables	Utilization status		Crude OR (95% CI)	Adjusted OR(95% CI)
	Yes	No		
**age of the respondent**				
≤19	79	168	1.7(1.13,2.63)	**4.750(.953, 23.676)**
>19	64	79	1.00	1.00
**Marital status of the respondent**				
Married	61	30	1.00	1.00
Not married	94	206	4.46(2.70,7.35)	0.852(.376, 1.930)
**Religion of the respondent**				
Orthodox	194	220	1.00	1.00
Others	6	16	1.81(.69,4.72)	**5.251 (1.032, 26.728)^+^**
**Schooling status**				
Currently student	65	191	5.88(3.73, 9.27).	**0**.**385 (1.032, 26.7)**
Currently not student	90	45	1.00	1.00
**Educational status of the respondent**				
Cannot read and writ	14	9	1.00	1.00
Can read and write	39	50	1.99(0.78,5.09)	1.4 (0.35,5.84)
1-8	29	14	0.75(0.26,2.15)	0.411 (0.08,2.15)
Grade 9 & above	73	163	3.47(1.44,8.389)	0.700 (0.17,2.93)
**Mother’s educational status**				
Cannot read and write	57	59	1.00	1.00
Can read and write	67	95	1.02(0.64,1.62)	0.837 (.398, 1.759)
1-8	21	17	0.58(0.28,1.21)	**0.224 (.068, .739)^+^**
Grade 9& above	10	43	3.103(1.44,6.69)	0.623 (.168, 2.315)
**Source of information Use TV**				
Yes	37	51	2.07(1.34,3.20)	1.458 (0.693, 3.068)
No	85	100	1.00	1.00
**Use phone**				
Yes	15	24	2.13(1.41,3.22).000	**2.625 (1.373, 5.016)^+^**
No	18	61	1.00	1.00
**Ever discussed about FP service**				
Yes	139	155	1.00	1,00
No	16	81	4.5(2.53,8.13)	**5.074 (2.070, 12.440)**
**Ever had sexual intercourse**				
Yes	108	21	1.00	1.00
No	47	215	23.5(13.4,41.35).000	**16.320 (6.27, 42.48)**

+ Significantly associated at p<0.05 %

## Discussion

In this study nearly all respondents (93.9%) knew about RHS which in line with a previous community-based study conducted in Jimma Town, South West Ethiopia [9]. The most-used reproductive health institutions were health centres followed by health posts and this finding is consistent with previous research that concluded the main health institutions are government health institutions (hospitals, health centres, and health posts) for RHS [9, 10].

Approximately a third of the respondents engaged in sexual intercourse, which was similar to use of RHS, and is consistent with other community-based cross-sectional research findings conducted in North West and South West Ethiopia [9, 11]. Those who do not ever have sexual activity are more likely to use services than whoever had. This is different from study done in 5747 teenagers aged 15 to 16 years in 25 schools in UK to [12]. This may be due to differences in the service delivery strategies in which in our case services are given most of the time with campaign rather than individuals use when they need it or it may be due to differences in safe sex practice (low safe sex practice in our community).

Parental monitoring, in the form of families asking their children about friends, was significantly associated with higher service utilization than families who didn’t ask, while participants with families that only asked sometimes were less likely to use services than with families who always ask. This is in line with studies done UK and Jimma [12, 13]. Participants with more parental monitoring may have better discussions on sexual issues and those who fear their parents may be more cautious to avoid any sexual related risks. Those who knew where RHS were provided were significantly more likely to utilize RHS and participants who utilized newspapers as source of information were also more likely utilize youth RHS than participants with less knowledge or those that did not use newspapers; these results are comparable with a study done in the UK [12].

FP utilization services were utilized by 40% of participants, which is consistent with the findings from Namibia (40%), Nigeria (45%), Kazakhstan (38%), and Kenya (46.7%) [14]. However, the finding in this study was higher than a study conducted in Asia (28%) and lower than the studies conducted in Brazil and England (62.1% and 89.2%, respectively) [15, 16]. A possible reason for the difference could be that the Brazil and England studies only included sexually-experienced adolescents, whereas the current study included both sexually-experienced and inexperienced adolescents. The Asia study only assessed family planning utilization during the first sexual intercourse, but the current study assessed family planning use at any time. The other differences might be the nature of study participants; the studies conducted in England and Brazil only included students who may have more knowledge and better attitudes towards using FP, but the current study included both students and adolescents who were out of school. In addition, the rural area youths might have poor knowledge and attitudes towards FP utilization.

Participants with younger fathers and mothers used FP more than those with older parents; this is in line with studies conducted in East Gojjam zone and the UK [17, 18]. Current students were also more likely to use FP than those out of school; this is comparable to a study conducted in UK. Those with mother’s educational status grade 1-8 were more likely to utilize FP than whose mothers cannot read and write; this is similar from studies done in USA and in East Gojjam zone [17]. Those who used phones as source of information were more likely to use FP services than those not use phones; this is in line with a study done in Kenya [18].

The overall prevalence of VCT utilization was 72.2%. This figure is considerably higher than the EDHS 2005 report in Ethiopia, in which only 14% of youth had utilized the service [19]. This may also be the participants in our study area may have better access and knowhow than youths at each corners of the country. The figure is also considerably higher compared with only 7.1% of Nigerian youth who had utilized the service [20]. The differences may be varying educational status, availability of VCT services, existence of awareness creation campaigns, and an overall difference in VCT strategic plan (in Ethiopia, VCT services are provided by Campion).

Adolescents 19 years and younger were more likely to use VCT services than those age 20-24 years. Participants with mothers educational status grade 9 and above were more likely to use VCT than those whose mothers cannot read and write; this is comparable with a study conducted in the USA [14]. Those who had never discussed VCT services were significantly more likely use the service than those who had discussed. This is lower than study done in Zambia [21]. In our study area there is a gap continuity of discussion, communication, or information to bring behavioral change. Adolescents who have information and have discussed VCT with different individuals may not think they need VCT service because they perceive a low risk.

A relatively high prevalence of self-reported STIs was observed among youths (35.5%), which is considerably higher than the study done in Woilayita Sodo University (23.3%) [22]. The difference might be due to the difference between study participants; university students have better knowledge about STIs, while in the current study, participants may have reported urinary tract infections as an STI.

Mothers’ educational status was positively associated with STI service utilization; this is consistent with a study done in the USA [14]. Participants who had more information about STIs, particularly those who had discussed STIs with their teachers were more likely to use STI services than their counter parts; this is similar to a community-based cross-sectional study conducted in Jimma [23].

The limitation of this study is it focuses on some components of youth reproductive health services (FP, STI & VCT) while others components are not addressed in this study.

## Conclusion

The authors conclude that, youth have ample information about reproductive health services, but in terms of use of reproductive health services, it is far behind from the expected level. Therefore, it is critical for programmers to look at beyond information delivery and creating awareness for young people. Sexual intercourse, parental monitoring, know where service provided, fathers educations, risk vulnerability and use of newsletter were found independent predictors for the RHS utilizations. Hence, building life skill in the way of positive attitude and skill for the service utilization, facilitating parent to child communication, establishing and strengthening of youth centres and increasing awareness for youth about those services are important steps to improve adolescents' reproductive health service utilization. Special focus needed in increasing youth friendly services, holding life skill trainings before the youth engage to risky behaviour; depth training particularly on RH service utilization and safe sex practice; and parents should monitor and teach their children, practice open discussion at the household about sexual health. The governmental and nongovernmental organizations should also target SRH trainings at the family level.

What is known about this topic

- Reproductive health programmes usually fill gaps to serve primarily urban youths, in which many of whom are also enrolled in formal schooling. And the vast majority of young people in the countryside remain underserved;
- Limited access to RH care and services for young people contributes to and exacerbates many of the RH problems like unwanted pregnancy and related complications, HIV and other STIs;
- Even if health services including RH services are accessible and acceptable, not all groups of youths can obtain the health services as they need.

What this study adds

- Though youth have information about reproductive health services, nearly half of youths are not using RH service in contrary to the information;
- Knowledge/information alone doesn’t help in reverting the negative outcomes of youths where huge investment in going on in the country.
